# Coping with the economic burden of non-communicable diseases among hypertensive and diabetic patients in private and public health facilities in Ado-Ekiti, Nigeria

**DOI:** 10.4314/gmj.v57i3.9

**Published:** 2023-09

**Authors:** Tope M Ipinnimo, Motunrayo T Ipinnimo, Ayodele K Alabi, Taiwo H Buari, Esther O Ajidahun, Olanrewaju K Olasehinde, Oluwadare M Ipinnimo, John O Ojo

**Affiliations:** 1 Community Medicine Department, Federal Teaching Hospital, Ido-Ekiti, Nigeria; 2 NHIS Department, Ekiti State University Teaching Hospital, Ado-Ekiti, Nigeria; 3 Medicine Department, Federal Teaching Hospital, Ido-Ekiti, Nigeria; 4 Medicine Department, Wesley Guild Hospital Ilesha Annex, Obafemi Awolowo; 5 University Teaching Hospital Complex, Ile-Ife, Nigeria; 6 Obstetrics and Gynaecology Department, Ekiti State University Teaching Hospital, Ado-Ekiti, Nigeria

**Keywords:** Coping strategies, economic burden, health facilities, Nigeria, non-communicable diseases

## Abstract

**Objective:**

To assess and compare how private and public health facilities patients cope with the economic burden of non-communicable diseases.

**Design:**

Comparative cross-sectional study.

**Setting:**

Thirty-nine private and eleven public health facilities in Ado-Ekiti, Nigeria

**Participants:**

Three hundred and forty-eight (Private:173; Public:175) patients with hypertension or diabetes, or both were recruited.

**Main Outcome Measures:**

Specific coping methods and numbers of coping strategies used by participants, as well as the perceived ability of participants to cope with the economic burden of non-communicable diseases.

**Results:**

Majority of participants paid through out-of-pocket (OOP) than through health insurance(HI) (Private:OOP:90.2% HI:9.8%; Public:OOP:94.3% HI:5.7%; p=0.152). More participants in private used instalment payments(p<0.001). However, other coping strategies showed no significant difference in both groups(p>0.05). Delayed treatment (Private:102; Public:95) was the most used strategy in both arms, and the number of strategies used by the participants showed no significant difference(p=0.061). Lower levels of education, out-of-pocket payment, increasing number of clinic visits, and hospital admission were associated with the use of higher numbers of coping strategies in both groups while being female and retired/unemployed were associated with the private arm.

**Conclusion:**

Although most patients in both groups pay out-of-pocket and use detrimental coping strategies, more patients in private arm use instalment payment, a non-detrimental method. Healthcare providers, especially public providers, should adopt policies encouraging patients to use non-detrimental coping strategies to meet their healthcare expenditures.

**Funding:**

None declared

## Introduction

Non-communicable diseases (NCDs) have replaced infectious diseases, malnutrition and morbidity associated with pregnancy and childbirth as the dominant public health problems.^1^ Globally, an estimated 41 million people die yearly due to NCDs, accounting for 74% of all deaths.^2^ Hypertension and diabetes are among the leading cause of the burden of NCDs, and they have both been shown to contribute significantly to mortality in sub-Saharan Africa.^3^,^4^ Hypertension is the commonest co-morbidity of diabetes and vice versa.^5^ Both conditions have emerged as a major public health problem and exert a huge financial burden on individuals, families, communities and any country's health system.^5^ The care of these diseases requires long-term financing. In Nigeria, this financing is borne almost entirely by individuals as the healthcare financing method is still mostly out-of-pocket.^6^

The out-of-pocket payment can exceed a patient's income and capacity to pay, sometimes triggering the use of coping strategies. These strategies are short-term methods used to meet the economic burden of illness.^7^ Some include selling assets, using treatment from cheaper alternatives at the expense of good quality ones, fund reallocation from food and investments, installment payment, borrowing, taking up other pieces of job, skipping appointments, and begging on the streets.^8^ While these strategies can help to cope with the immediate economic burden, they can also leave the household at risk of long-term poverty.^9^ There is evidence that health expenditure exceeding a certain threshold of a household's ability to pay may lead to catastrophic expenditure and medical poverty trap.^10^

Coping strategies could be detrimental when they draw on individuals' ability to create income, leave them indebted, or put them at increased risk of catastrophic expenditure and poverty.^11^ Examples of non-detrimental strategies include using social networks and social capital, instalment payment, and labour substitution. In contrast, detrimental coping strategies include borrowing/loan, abandonment and delayed treatment, and selling off assets meant for livelihood.^9^ Non-detrimental coping strategies can help prevent catastrophic health expenditure and impoverishment.

In Kyrgyzstan, 4% of patients do not use coping strategies, while more than half (56%) use two or more strategies.^9^ The choice of a coping strategy differs and usually depends on a household's asset base.^7^ Savings (67%), social welfare (56%) and support from social networks (35%) were the most commonly used strategies.^9^ A study among diabetes patients in Southeastern Nigeria showed that the use of savings (99%), support from family members (85%) and a donation from friends (55%) were the most common methods of coping with payment, while government waiver(1%) and health insurance (3%) were the least used.^7^ Another study among diabetic and hypertensive patients in South-South, Nigeria found that 4.2% used the prepayment method. In contrast, the rest had to pay at the point of access with their own money or assistance from others.^4^

Borrowing was found to be the most used (45%) coping strategy among NCD patients in Southwest Nigeria, and of the lenders, friends (54.5%) and family members (19.2%) constituted the commonest.^12^ This coping method is usually combined with at least two or three additional strategies.^9^ Borrowing, in addition to strategies such as interruption of children's education, skipping appointments and community-based support, occurred most among the poorest households.^7^

Similarly, it has been published that individuals with low income and high health expenditure were more likely to use detrimental coping methods.^11^

Furthermore, only about one-quarter of patients with NCDs in Nigeria find it easy to cope with their healthcare payments, while others reported different degrees of difficulty.^4^ This research presents new and vital information about how patients cope with the economic burden of these diseases in private and public health facilities in Nigeria. This study identifies the gap between the coping methods employed by NCD patients in private and public health facilities. Research on coping with the financial burden of these chronic conditions is imperative as they require long-term treatment and access to healthcare, resulting in a constant need for money to cover the payment, especially in a country with most of its population paying for healthcare out-of-pocket. This study's findings will help enrich the literature and assist policymakers in formulating healthcare policies that will assist patients with these diseases to cope with their healthcare payments. This study assesses and compares how hypertensive and diabetic patients cope with the economic burden of NCDs in private and public health facilities in Ado-Ekiti, Nigeria. The scope of NCDs studied was limited to hypertension and diabetes, Nigeria's leading causes of morbidity and mortality.^4^

## Methods

### Study design, setting and participants

A comparative cross-sectional study was carried out in Ado-Ekiti, Nigeria. Ado-Ekiti is bounded to the north by Irepodun/Ifelodun Local Government, to the south by Ise/Orun and Ikere Local Governments, to the east by Ekiti Southwest Local Government and the west by Gbonyin Local Government. The people of Ado-Ekiti are mainly of the Ekiti sub-ethnic group of the Yorubas, with a population of 308,621^13^ as of 2006 (464,795 in 2019 projected population). It was estimated that 38% and 5% of adults in the city are hypertensive and diabetic, respectively.^5^ Hospital morbidity and mortality in Ado-Ekiti result from hypertension, diabetes and complications.^14^ There is no special program by the State Government targeted at only these diseases. However, through the State Ministry of Health, the State Government carries out free medical outreach services once a year to communities within the city and its environs to cater to the medical needs of the people. Non-governmental organizations such as Mercy International, Development Social Institute and religious bodies also carry out similar medical outreaches. This initiative might be able to cater to some disease conditions but will not be adequate for NCDs where regular follow-up is important.

This study was carried out between October and November 2019 among adult patients with hypertension or diabetes. Patients with hypertension or diabetes, or both more than 18 years of age, who had been on treatment for their health condition for at least three consecutive months before the study were included, while those that were pregnant or too ill to respond were excluded from the study.

### Sample size calculation and sampling technique

After using the formula for a comparative study,^15^ an adequate sample size of 360 patients (180 in each group) was calculated based on assumptions of 95% confidence level, 90% power, 10% adjustment for non-response and the economic burden of illnesses in a private and public health facility in a previous study.^16^

About a third of the health facilities in Ado-Ekiti were randomly selected (11 out of the 30 public health facilities and 39 out of the 108 private health facilities) for this study. The private health facilities operate a near-similar protocol. This is the same for the public health facilities except for the teaching hospital with more staffing, highly skilled professionals, diagnostic tests and equipment with bureaucracy. The number of patients selected per health facility in each group was based on proportionate allocation using the number of patients with hypertension and diabetes seen in a month. At the health facilities, the list of patients in the clinic registers was used as the sampling frame. A systematic sampling technique was used to select eligible patients immediately after their clinic consultation. A total of 348 (173 in private; 175 in public) patients with hypertension or diabetes, or both with complete data, were eventually recruited.

### Data collection

Data were collected with a semi-structured questionnaire adapted from that used in previous studies.^4^,^7^ The questionnaire elicited information on socio-demographic characteristics, specific coping methods, and their perceived ability to cope with the economic burden of NCDs. Pre-testing of the questionnaire was done on 36 subjects in private and public health facilities in Akure, a nearby city. The questionnaire was translated into the local language (Yoruba) for field use and back-translated into English with Yoruba linguists from the Ekiti State University, Ado-Ekiti, to ensure correctness and consistency of meaning. Appropriate corrections were made to the questionnaire after pre-testing where necessary.

### Data analysis

Data were entered and analyzed with computer software, IBM SPSS Statistics for Windows, Version 22.0 (IBM Corp., Armonk, N.Y., USA). Sex, payment methods, other categorical socio-demographic data, patients' coping strategy, and financial coping ability were summarized using frequency and percentages. Mean, and standard deviation was used to summarize the age of participants. The income was categorized into <₦30,000 and ≥₦30,000 based on Nigeria's minimum wage (₦30,000; $83.33) as of 2019. The mean age of the patients was compared between private and public health facilities using the independent Student-t-test. The Chi-square test was used to assess if there was a statistically significant difference in the distribution of socio-demographic variables and coping strategies in the two groups. Additionally, the association between payment method and financial coping ability of the participants as well as the number of coping strategies and socio-demographic variables, was determined with the Chi-square test. The level of statistical significance was set at p < 0.05.

### Ethical consideration

Ethical approval (Reference Number: ERC/2018/10/03/145A) was obtained from the Ethics and Research Committee of the Federal Teaching Hospital, Ido-Ekiti, Nigeria. All respondents gave informed consent, and the study was conducted according to the Helsinki Declaration principles.

## Results

Table 1 shows the socio-demographic characteristics of the participants. The mean age ± standard deviation of the participant was 59.5 ± 10.5 years in private health facilities and 59.7 ± 11.2 years in public health facilities (p = 0.910). Most participants pay out-of-pocket in both groups (Private, 90.2%; Public, 94.3%; p = 0.152). There was a statistically significant difference in the income (p = 0.013) and level of education (p = 0.003) of the participants in the two groups. Those in private health facilities earn more and had a higher level of education than those in public health facilities.

**Table 1:** Socio-demographic characteristics of the participants

Variables	Health Facility		Test	p-value
	Private (%), n= 173	Public (%), n= 175		
***Mean age ± S.D. (Years)***	*59.51 ± 10.49*	*59.65 ± 11.21*	*−0.113* ^T^	*0.910*
**Sex**				
**Male**	78 (45.1)	79 (45.1)	<0.001^X^	0.992
**Female**	95 (54.9)	96 (54.9)		
**Level of education**				
**No formal education**	7 (4.0)	20 (11.4)	13.807^X^	**0.003**
**Primary education**	27 (15.6)	38 (21.7)		
**Secondary education**	52 (30.1)	58 (33.2)		
**Tertiary education**	87 (50.3)	59 (33.7)		
**Marital status**				
**Unmarried** ^#^	52 (30.1)	50 (28.6)	0.093^X^	0.761
**Married**	121 (69.9)	125 (71.4)		
**Religion**				
**Christianity**	135 (78.0)	128 (73.1)	1.128^X^	0.288
**Islam**	38 (22.0)	47 (26.9)		
**Tribe**				
**Yoruba**	142 (82.1)	136 (77.7)	1.262^X^	0.738
**Hausa**	6 (3.4)	9 (5.1)		
**Igbo**	14 (8.1)	18 (10.3)		
**Others (Ebira, Tiv, Efik)**	11 (6.4)	12 (6.9)		
**Occupation**				
**Formal**	59 (34.1)	48 (27.4)	2.767^X^	0.251
**Informal**	76 (43.9)	92 (52.6)		
**Retired/Unemployed**	38 (22.0)	35 (20.0)		
**Income**				
**<₦30,000 ($83.33)**	42(24.3)	64(36.6)	6.208^X^	**0.013**
**≥₦30,000 ($83.33)**	131(75.7)	111(63.4)		
**Payment Method**				
**Health Insurance**	17 (9.8)	10 (5.7)	2.056^X^	0.152
**Out of pocket**	156 (90.2)	165 (94.3)		
**Number of Clinic Visits in the last 1 month**				
**0**	5 (2.9)	5 (2.9)	5.830^X^	0.054
**1**	134 (77.4)	116 (66.2)		
**2 or more**	34 (19.7)	54 (30.9)		
**Number of Admissions in the last 1 month**				
**0**	155 (89.6)	158 (90.3)	0.046^X^	0.830
**1**	18 (10.4)	17 (9.7)		

X Chi-square test

T T-test, SD—Standard deviation

# (Unmarried included-Single/Divorced/Widowed)

Table 2 shows the coping strategies used by participants and their ability to cope with the financial burden of NCDs. There was a statistically significant difference in the use of instalment payment as a coping strategy by participants, with more participants in private using it than in public health facilities (p < 0.001).

**Table 2:** Coping strategies used by the participants and ability to cope with the financial burden of NCDs

Variable	Health Facility		X^2^	p-value
	Private (n=173)	Public (n=175)		
**Coping Strategies** ^**MR**^				
**Delayed treatment** ^**D**^	102 (51.8)	95 (48.2)	1.149	0.284
**Loan or borrowing from bank/cooperative/friends/family** ^**D**^	62 (48.4)	66 (51.6)	0.132	0.717
**Personal savings** ^**D**^	62 (50.4)	61 (49.6)	0.037	0.848
**Fund reallocation from food** ^**D**^	62 (52.1)	57 (47.9)	0.413	0.521
**Abandoned treatment** ^**D**^	39 (52.7)	35 (47.3)	0.336	0.562
**Use of treatment from cheaper alternative** ^**D**^	37 (56.9)	28 (43.1)	1.662	0.197
**Sold properties/ Assets** ^**D**^	9 (47.4)	10 (52.6)	0.044	0.834
**Fund reallocation from children school fees** ^**D**^	6 (66.7)	3 (33.3)	0.480^Y^	0.488
**Street begging/In-kind payment/money from business** ^**D**^	3 (50.0)	3 (50.0)	<0.001^Y^	0.999
**Installment payment** ^**ND**^	42 (80.8)	10 (19.2)	23.587	**<0.001**
**Taking up other pieces of jobs** ^**ND**^	9 (47.4)	10 (52.6)	0.044	0.834
**Donations and gifts** ^**ND**^	12 (70.6)	5 (29.4)	3.116	0.078
**Numbers of coping strategies**				
**None**	21 (12.1)	18 (10.3)	10.573	0.061
**1**	23 (13.3)	27 (15.4)		
**2**	50 (29.0)	65 (37.2)		
**3**	39 (22.5)	46 (26.3)		
**4**	22 (12.7)	10 (5.7)		
**5 or more**	18 (10.4)	9 (5.1)		
**Ability to cope with the financial burden of NCDs**				
**Very easy**	8 (4.6)	8 (4.6)	1.795	0.616
**Easy**	18 (10.4)	13 (7.4)		
**Difficult**	126 (72.8)	126 (72.0)		
**Very difficult**	21 (12.2)	28 (16.0)		

X^2^-Chi-square test, ^Y^-Continuity correction, ^M.R.^—Multiple responses, ^D^--Detrimental coping strategies ^N.D.^--Non-detrimental coping strategies

However, other strategies showed no significant difference in the proportion of participants using them in private and public health facilities (p > 0.05). Delayed treatment was the most used coping strategy, with 102 and 95 participants using them in private and public health facilities, respectively. Loan/borrowing (Private, 62; Public, 66) and personal savings (Private, 62; Public, 61) were the second and third most used strategies. The least used strategy was street begging/in-kind payment/money from business (Private, 3; Public, 3).

There was no significant difference in the number of strategies used by the participants (p = 0.061). About a tenth (Private, 12.1%; Public, 10.3%) of the participants did not use any coping strategy, while most combined at least two strategies. A minority of the patients (Private, 4.6%; Public, 4.6%) agreed that coping ability with the financial burden of NCDs was very easy, while most (Private, 72.8%; Public, 72.0%) described it as difficult. Nonetheless, there was no statistically significant difference in the description of the patient's coping ability with the financial burden of NCDs in the two groups (p = 0.616).

Table 3 shows that the level of education (Private, p = 0.009; Public, p < 0.001), payment method (p<0.001), number of clinic visits (Private, p = 0.010; Public, p = 0.025) and hospital admission in the last one month (Private, p = 0.037; Public, p = 0.040) were significantly associated with the number of coping strategies in the two groups. Sex (p=0.024) and occupation (p<0.001) were also associated with private health facilities.

**Table 3:** Association between the number of coping strategies and socio-demographic variables of the participants

Variable	Health Facility							
	Private (n= 173)				Public (n= 175)			
	Numbers of coping strategies			X^2^ (p-value)	Numbers of coping strategies			X^2^ (p-value)
	**0** ***n*=21**	**1** **n=23**	**>1** ***n*=129**		**0** ***n*=18**	**1** ***n*=27**	**>1** **n=130**	
**Sex**								
**Male**	14 (17.9)	6 (7.7)	58 (74.4)	7.304	10 (12.7)	11 (13.9)	58 (73.4)	1.014
**Female**	7 (7.4)	17 (17.9)	71 (74.7)	**(0.024)**	8 (8.3)	16 (16.7)	72 (75.0)	(0.609)
**Level of education**								
**No formal education**	0 (0.0)	3 (42.9)	4 (57.1)	15.697^F^	0 (0.0)	4 (19.0)	17 (81.0)	24.575^F^
**Primary education**	0 (0.0)	7 (25.9)	20 (74.1)	**(0.009)**	3 (7.9)	2 (5.3)	33 (86.8)	**(<0.001)**
**Secondary education**	6 (11.5)	3 (5.8)	43 (82.7)		1 (1.8)	8 (14.0)	48 (84.2)	
**Tertiary education**	15 (17.2)	10 (11.5)	62 (71.3)		14 (23.7)	13 (22.0)	32 (54.2)	
**Marital status**								
**Unmarried#**	6 (11.5)	6 (11.5)	40 (76.9)	0.250	6 (12.0)	7 (14.0)	37 (74.0)	0.293
**Married**	15 (12.4)	17 (14.0)	89 (73.6)	(0.926)	12 (9.6)	20 (16.0)	93 (74.4)	(0.889)
**Occupation**								
**Formal**	17 (28.8)	10 (16.9)	32 (54.2)	34.895^F^	8 (16.7)	10 (20.8)	30 (62.5)	5.969^F^
**Informal**	0 (0.0)	12 (15.8)	64 (84.2)	**(<0.001)**	8 (8.7)	14 (15.2)	70 (76.1)	(0.194)
**Retired/Unemployed**	4 (10.5)	1 (2.6)	33 (86.8)		2 (5.7)	3 (8.6)	30 (85.7)	
**Income**								
**<₦30,000 ($83.33)**	0 (0.0)	6 (20.7)	23 (79.3)	5.767^Y^	1 (2.0)	9 (18.0)	40 (80.0)	5.280^Y^
**≥₦30,000 ($83.33)**	21 (14.6)	17 (11.8)	106 (73.6)	(0.051)	17 (13.6)	18 (14.4)	90 (72.0)	(0.075)
**Payment Method**								
**Health Insurance**	17 (100.0)	0 (0.0)	0 (0.0)	136.457^Y^	9 (90.0)	1 (10.0)	0 (0.0)	73.604^Y^
**Out of pocket**	4 (2.6)	23 (14.7)	129 (82.7)	**(<0.001)**	9 (5.5)	26 (15.8)	130 (78.8)	**(<0.001)**
**Number of Clinic Visits in the last 1 month**								
**0**	0 (0.0)	3 (60.0)	2 (40.0)	11.922^F^	2 (40.0)	0 (0.0)	3 (60.0)	10.229^F^
**1**	20 (14.9)	18 (13.4)	96 (71.6)	**(0.010)**	14 (12.1)	22 (19.0)	80 (69.0)	**(0.025)**
**2 or more**	1 (2.9)	2 (5.9)	31 (91.2)		2 (3.7)	5 (9.3)	47 (87.0)	
**Number of Admissions in the last 1 month**								
**0**	21 (13.5)	23 (14.8)	111 (71.6)	6.853^Y^	18 (11.4)	27 (17.1)	113 (71.5)	6.518^Y^
**1**	0 (0.0)	0 (0.0)	18 (100.0)	**(0.037)**	0 (0.0)	0 (0.0)	17 (100.0)	**(0.040)**

X^2^- Chi-square test, ^Y^-Continuity correction, ^F^-Fisher's exact test, #--(Unmarried included-Single/Divorced/Widowed)

Lower levels of education, out-of-pocket payment, and an increasing number of clinic visits and hospital admission were associated with using higher numbers of coping strategies in both groups. In addition, being female and retired/unemployed was associated with using more coping strategies in private health facilities., males, patients- with tertiary education, in formal jobs, with health insurance, with 1 clinic visit. Those without admission in the last month used no coping strategy in private health facilities. In contrast, patients with tertiary education, health insurance, no clinic visit, and no admission in the last Table 4 shows the association between payment methods and the ability to cope with the financial burden of NCDs. In both groups, payment with health insurance was associated with a very easy or easy ability to cope, while payment out-of-pocket was associated with a difficult and very difficult ability to cope with the financial burden of NCDs (Private, p < 0.001; Public, p < 0.001).

**Table 4:** Association between payment method and financial coping ability of the participants

Variable	Health Facility							
	Private (n= 173)				Public (n= 175)			
	Payment Method		X^2^	p-value	Payment Method		X^2^	p-value
	Health Insurance (*n*=17)	Out-of-pocket (*n*=156)			Health Insurance (*n*=10)	Out-of-pocket (*n*=165)		
**Ability to cope with the financial burden of NCDs**								
**Very easy**	8 (47.1)	0 (0.0)	79.053^f^	**<0.001**	7 (70.0)	1 (0.6)	52.694^f^	**<0.001**
**Easy**	9 (52.9)	9 (5.8)			3 (30.0)	10 (6.1)		
**Difficult**	0 (0.0)	126 (80.8)			0 (0.0)	126 (76.4)		
**Very difficult**	0 (0.0)	21 (13.4)			0 (0.0)	28 (16.9)		

f Fisher's exact test

## Discussion

This study compared how patients cope with the economic burden of NCDs in private and public health facilities. In this study, the majority of participants pay out-of-pocket in both groups. More participants in private use instalment payments as a coping strategy than in public health facilities. However, other coping strategies showed no significant difference. Delayed treatment, loan/borrowing, and personal savings were the most used strategies in the two groups.

Furthermore, most participants described their financial coping as difficult, associated with out-of-pocket payments in private and public health facilities. Lower levels of education, out-of-pocket payment, and the increasing number of clinic visits and hospital admission were associated with using higher numbers of coping strategies in both groups.

In addition, being female and retired/unemployed were associated with using more coping strategies in private health facilities. The findings on high out-of-pocket payment are similar to previous studies done among patients with hypertension and diabetes in Nigeria.^4^,^17^ The high prevalence of this payment method may be due to the low coverage of suitable prepayment alternatives.^7^,^18^,^19^

Patients with NCDs utilize different coping strategies to meet up with out-of-pocket healthcare payments. The strategies for coping with this economic burden can indicate how well the social protection system in a country functions and whether adjustments are necessary.^9^ Delayed treatment was the most used strategy, followed by loan/borrowing and personal savings in private and public health facilities. This finding differs slightly from what was found in Kyrgyzstan among patients with diabetes or tuberculosis, where income/savings and social welfare/donations were the most used financial coping strategies.^9^

Nevertheless, skipping appointments or delayed treatment has been reported in studies among patients in West Africa as a coping method used to meet up with the economic burden of care.^7^,^20^ However, this strategy could result in these patients coming down with acute and chronic complications, which will require additional interventions and sometimes hospitalization leading to higher healthcare expenditure.

Furthermore, borrowing has been reported as a major coping mechanism used to meet the economic burden of care among patients.^7^,^12^ About 30% of households in West Africa use this strategy.^20^ However, it was the least-used method among patients with diabetes, tuberculosis, or both in Kyrgyzstan.^9^ Borrowing is associated with decreasing wealth and may attract a high-interest rate, especially from banks and other professional money lenders.^20^ In addition, it may require collateral, usually in the form of an individual or household asset. High-interest rate and collateral could be detrimental as it draws on patients' ability to generate future income. It leaves patients indebted and thus at higher risk of catastrophic health expenditure and poverty.^21^

Personal saving was also identified as one of this study's most used coping strategies. Incomes and savings have been reported as a popular coping strategy in sub-Saharan Africa, and about 40% of individuals in this region cope with healthcare payments through it.^20^ A study in Southeast Nigeria reported savings as the most used strategy to cope with healthcare costs.^7^ However, savings was not identified as a coping strategy in a study among chronic disease patients in Southwest Nigeria.^12^ Using savings as a coping strategy is likely to increase the economic burden of NCDs, especially on the poorest individuals.^7^ When money saved for basic needs such as food and shelter is mobilized to pay for medical bills, there could be a reduction in the consumption of such items, which are important for health and well-being. This may deteriorate patients' well-being and health, further impoverishing them.

This study also revealed that many participants in private health facilities use instalment payments as a coping mechanism. This may result from the bureaucracy in public health institutions, especially when the decision to pay in instalments would have to pass through several desks before the final approval. This decision chain is shorter in most private health facilities where the head of the facility that makes such a decision is usually the physician treating the patient. It may also suggest that private health facilities may be more flexible and lenient with payment by allowing patients to pay in bits and at ease. Instalment payments have been reported as a coping strategy among patients in Nigeria.^8^ Paying medical bills in instalments may be convenient and could ease the economic burden of NCDs care. Payment coping strategies like this could be very important, especially in times of sudden financial stress, such as hospital admission and surgical interventions where the full payment may not be available at once. This and other non-detrimental strategies could help alleviate the economic impact of healthcare expenditure.^9^ Unfortunately, only a few patients use them, particularly in public health institutions. Also, formal health insurance, which could help minimize healthcare costs, is lacking,^4^,^12^ making the patients bear the full brunt of the economic impact of care. These economic effects have significant implications for healthcare policy in the country.

In terms of the ability to cope with financing NCDs care expenditures, most participants admitted facing some difficulties in meeting up. This situation is not different as a previous study revealed that three-quarters of patients with NCD agreed to have varying degrees of difficulties in meeting the financial requirement of their conditions.^4^ The reason for this may be because payments were made mostly out-of-pocket. This form of payment has been invariably recognized as retrogressive, and many developed health systems have moved away from it to prepayment methods.^4^ Payment with health insurance was found to be significantly associated with ease of payment none of the participants on health insurance had any form of difficulty paying for their healthcare. Previous studies among NCD patients have shown that patients enrolled under health insurance are less likely to face financial difficulty when paying for care.^17^ This further emphasizes the need to continue advocating for the increased coverage of health insurance among patients with NCDs.

One limitation of this study is that the study's exploratory nature relied on self-reported data, which may be affected by recall biases. In addition, this research is health facility-based, capturing only patients seeking healthcare in health facilities. The study did not capture those who chose to cope with the economic burden by not seeking care at health facilities.

## Conclusion

This study provides valuable contribution on how patients cope with the economic burden of NCDs in Nigeria's private and public health facilities. The NCD patients pay mostly out-of-pocket and utilise financial coping strategies that could impair their ability to generate future income, leaving them indebted, and at a greater risk of poverty. Although a minority of them use non-detrimental coping methods especially those in the private health facilities, this could be improved upon and expanded to the public institutions.

Therefore, it is important for healthcare providers, especially public providers, to implement policies that will encourage patients to adopt non-detrimental coping strategies such as instalment payments to meet their healthcare expenditures. Also, expanding health insurance coverage among these patients would reduce the high out-of-pocket spending and its deleterious impacts.
